# Insula and somatosensory cortical myelination and iron markers underlie individual differences in empathy

**DOI:** 10.1038/srep43316

**Published:** 2017-03-03

**Authors:** Micah Allen, Darya Frank, James C. Glen, Francesca Fardo, Martina F. Callaghan, Geraint Rees

**Affiliations:** 1 Institute of Cognitive Neuroscience, UCL, Alexandra House, 17 Queen Square, London, WC1N 3AZ, UK; 2 Wellcome Trust Centre for Neuroimaging, UCL, 12 Queen Square, London, WC1N 3BG, UK; 3 Division of Neuroscience and Experimental Psychology, University of Manchester, 46 Grafton Street, Manchester, M13 9NT, UK.; 4 Danish Pain Research Centre, Department of Clinical Medicine, Aarhus University, Hospital, Norrebrogade 44,Building 1A, 1st floor, DK-8000 Aarhus C, Denmark; 5 Interacting Minds Centre, Aarhus University, 8000 Aarhus, Denmark

## Abstract

Empathy is a key component of our ability to engage and interact with others. In recent years, the neural mechanisms underlying affective and cognitive empathy have garnered intense interest. This work demonstrates that empathy for others depends upon a distributed network of regions such as the insula, parietal cortex, and somatosensory areas, which are also activated when we ourselves experience an empathized-with emotion (e.g., pain). Individuals vary markedly in their ability to empathize with others, which predicts the tendency to help others and relates to individual differences in the neuroanatomy of these areas. Here, we use a newly developed, high-resolution (800 μm isotropic), quantitative MRI technique to better elucidate the neuroanatomical underpinnings of individual differences in empathy. Our findings extend previous studies of the neuroanatomical correlates of cognitive and affective empathy. In particular, individual differences in cognitive empathy were associated with markers of myeloarchitectural integrity of the insular cortex, while affective empathy was predicted by a marker of iron content in second somatosensory cortex. These results indicate potential novel biomarkers of trait empathy, suggesting that microstructural features of an empathy and body-related network are crucial for understanding the mental and emotional states of others.

Empathy is a core social skill underlying our ability to understand and respond appropriately to the emotions of others. Following growing interest from social neuroscientists, the neural underpinnings of both trait and state empathy has become a topic of intensive research^1^,^2^,^3^. Healthy individuals differ considerably in their tendency and ability to empathize^4^,^5^, which in turn predicts real world behaviours such as charitable giving^6^,^7^,^8^, and is affected by genetic and environmental factors^9^. In general, variation in the macroscopic structure of the brain underlies individual differences in a variety of perceptual, emotional, and cognitive behaviours^10^. Here we use a recently developed quantitative MRI technique to examine the neurobiological factors underlying individual differences in cognitive and affective empathy, as captured by the Interpersonal Reactivity Index^4^ (IRI).

There is ample evidence relating individual differences in self-reported trait empathy to social behavior and implicit neural and physiological responses. For example, scores on the IRI correlate with the tendency to display bullying and defending behaviour^11^. Although the mapping between trait and state empathy is complicated^12^,^13^, several physiological studies have validated the IRI using implicit physiological measures. In one recent electromyography (EMG) study, participants with greater scores on the affective component of the IRI were more prone to frown when viewing another person in pain^14^. In another study^15^, scores on the affective sub-scale Personal Distress (PD) were correlated with skin conductance response (SCR) and the P300 ERP component in schizophrenic patients viewing others in pain. These and other studies point to a stable individual tendency to empathize with others, which also predicts the style and intensity of individual empathetic and emotional states.

Extending these findings, previous electrophysiological and functional imaging studies also correlated the IRI with brain responses to a variety of emotional and affective stimuli in healthy adults and patient populations (e.g., fMRI^16^,^17^,^18^,^19^,^20^, EEG^21^, and MEG^22^). For example, Singer *et al*.^23^ found that affective empathy scores (a subscale of the IRI) were positively correlated with BOLD responses to empathy-for-pain in the ACC and left insula. Another study showed positive correlations between affective empathy scores and activity elicited by watching another’s pain in the premotor and somatosensory cortices^24^. These studies thus highlight a distributed network of cortical areas involved in the experience of empathy.

Complementing these results, a number of recent studies investigated whether individual differences in self-reported trait empathy relate to local cortical grey matter volume, using voxel-based morphometry (VBM)^25^,^26^,^27^,^28^. These studies report, that affective empathy scores on the personal distress and empathic concern subscales of the IRI correlate negatively with grey matter volume in the somatosensory cortex, and positively in the insula^25^. The latter finding has been further replicated using other trait-empathy questionnaires^26^,^27^. While these studies point towards a potential neuroanatomical basis for individual differences in trait empathy, they are limited by the inherent neurobiological ambiguity of VBM. Although VBM and other similar techniques provide an initial insight into mesoscopic neuroanatomy, they are ambiguous as to the specific neurobiological factors driving changes in these measures^29^,^30^,^31^. For example, cortical volume and thickness measures are both impacted by variability in cortical folding, which in turn can be related to a non-specific variety of microstructural features^32^.

Recent developments in quantitative MRI techniques allow the direct mapping of specific MRI parameters that are sensitive to underlying myeloarchitecture, iron, and macromolecule content (e.g., myelination, oligodendrocytes and other support structures). This is a first step towards *in vivo* histology using MRI^33^. Group studies using voxel based quantification (VBQ)^34^ provide new insights into the neural mechanisms underlying individual differences in neural anatomy^35^,^36^, and can be directly compared across time points and imaging sites to facilitate clinical research^37^,^38^. Here our aim was to expand on previous studies that have investigated the relationship of brain volume and IRI-based measures of trait cognitive and affective empathy^25^,^26^,^27^,^28^, illuminating the neuroanatomical underpinnings of these effects. We thus employed the improved histological specificity of VBQ to map the microstructural correlates of cognitive and affective trait empathy.

## Results

### IRI Results

To facilitate comparison of our results with published norms and previous neuroanatomical studies of empathy, we first calculated descriptive statistics and correlations between the IRI subscales. IRI subscale scores were normally distributed and consistent with previously reported norms^39^. As in previous studies^25^, we found that scores on Empathic Concern correlated positively with the Perspective Taking (*r*(46) = 0.46, *p* < 0.001) and Fantasy (*r*(46) = 0.48, *p* < 0.001) subscales. We did not observe any other correlations across the other subscales. Additionally, as previously reported we found that Personal Distress was greater in females than in males (*t*(46) = −2.63, *p* = 0.012); no other gender differences were found. See Table 1 for an overview of all summary statistics, and Supplementary Tables 1 and 2 for summary correlations and t-tests.

### Voxel Based Quantification

**Figure 1:**
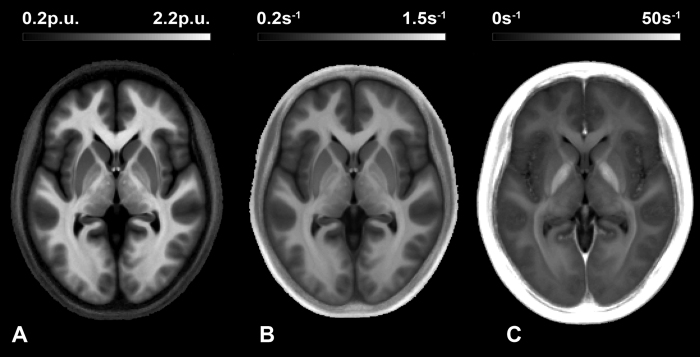
Example parameter maps used for voxel-based quantification analyses: magnetization transfer, MT (**A**); longitudinal relaxation rate, R_1_ (**B**); and transverse relaxation rate, R_2_* (**C**). Respectively, each map is primarily sensitive to macromolecular content and myeloarchitecture, myelination, and iron (note hyper-intensity in iron-rich midbrain structures). Average of 48 normalized maps presented at origin.

We first focused on extending previous findings, examining microstructural correlates of IRI subscales at coordinates previously related to trait-empathy. To do so, we conducted VBQ multiple regression analyses (see *VBQ Methods,* and Fig. 1), with each subscale predicting markers of local iron concentration (R_2_*)^40^ myelination (R_1_)^41^ or myeloarchitectural integrity (MT)^42^ within an *a priori* volume-of-interest (VOI) mask of regions previously reported to show anatomical covariation with trait empathy^25^,^26^,^27^,^28^. As the microstructural correlates identified by VBQ do not necessarily relate to macroscopic volume, we next repeated this analysis across the whole brain, correcting for multiple comparisons. All VBQ analyses were controlled for potential confounding variables of age, gender, total intracranial volume, and the gender by personal distress interaction (see *IRI Results*).

### VBQ Results – VOI Analysis

Our VOI analyses found that empathic concern (EC) positively correlated with R_1_ in the right inferior parietal lobule (IPL, peak MNI_xyz_ = 62, −59, 18, *p*FWE = 0.010, *T* = 5.57). R_1_ in the same region was also negatively related to the fantasy (FS) subscale (*p*FWE = 0.020, *T* = 5.32). R_2_* was negatively related to perspective taking in the right anterior insula (peak MNI_xyz_ = 37, 19, 5, *p*FWE = 0.042, *T* = 4.96). As these results suggest that the tendency to engage in Fantasy vs. Empathic Concern may depend on right IPL myelination, we calculated a difference score for each participant (i.e., FS – EC) and correlated it with R_1_ values extracted from our right peak region; indeed our difference score correlated negatively with R_1_ in this region (*r*(46) = −0.67, *p* < 0.001), confirming that individuals with a higher tendency to engage in Fantasy vs Empathic Concern exhibited greater IPL myelination. No other effects were observed at the chosen level of statistical significance (*p*FWE < 0.05) for any quantitative map or subscale. These results indicate that the individual trade-off between Fantasy vs Empathic Concern-based empathy is related to IPL myelination, whereas individuals who report a greater tendency to engage in perspective taking have a lower concentration of iron in the right anterior insula. Table 2A provides an overview of these results.

### VBQ Results – Whole Brain

**Figure 2:**
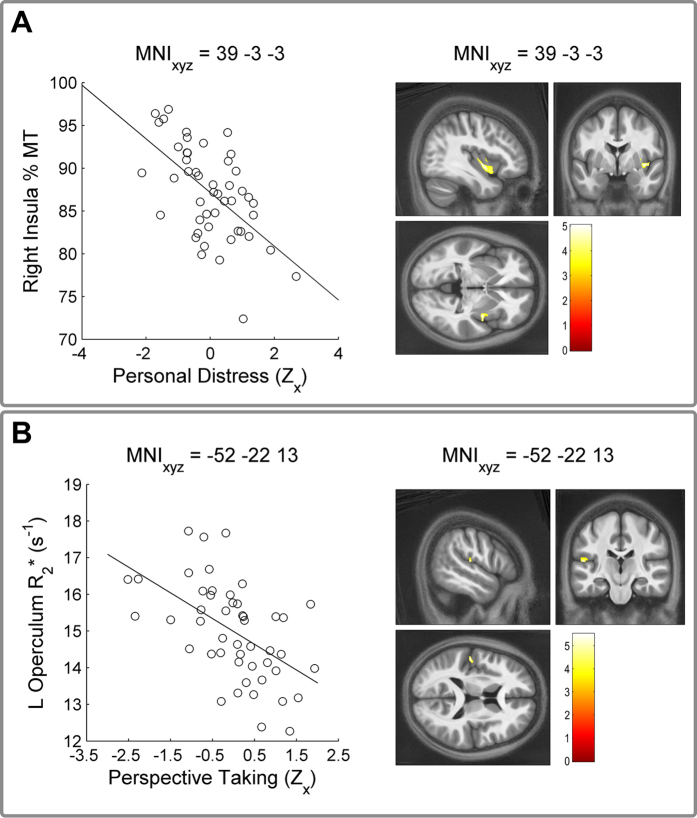
Results of Voxel-Based Quantification Analyses. Microstructural correlates (MT, R_1_, R_2_*) of each Inter-personal Reactivity Index subscale were analysed in multiple regression analyses controlling for age, gender, and total intracranial volume. (**A**) Affective empathy, as measured by the Personal Distress (PD) subscale negatively predicts markers of macromolecular myeloarchitecture in the right mid-insula. (**B**) Cognitive empathy (Perspective Taking, PT) instead negatively relates to iron content in the left operculum/SII. Whole-brain, non-stationarity corrected cluster-level inference, *p*FWE < 0.05. Statistical parametric maps presented on the average of all 48 normalized MT maps; colorbar indicates voxelwise t-value. For illustration purposes, scatter plots depict extracted signal at the peak coordinate vs each z-scored subscale, after nuisance variable correction.

Using a whole-brain, cluster-corrected threshold, we found that R_2_* in the left operculum/secondary somatosensory cortex (SII) negatively correlated with Perspective Taking (PT). In contrast, Personal Distress (a measure of affective empathy, PD) negatively correlated with MT in the right insula, in a cluster extending from the mid-to-anterior portion. PD was also negatively related to MT in the right caudate. These results suggest that reduced SII microstructural iron concentration is predicted by increased cognitive empathy whereas the tendency to experience negative emotions in response to other’s experiences relates to the myeloarchitecture of the insula and caudate. Figure 2 and Table 2B provide a summary of these results.

**Figure 3:**
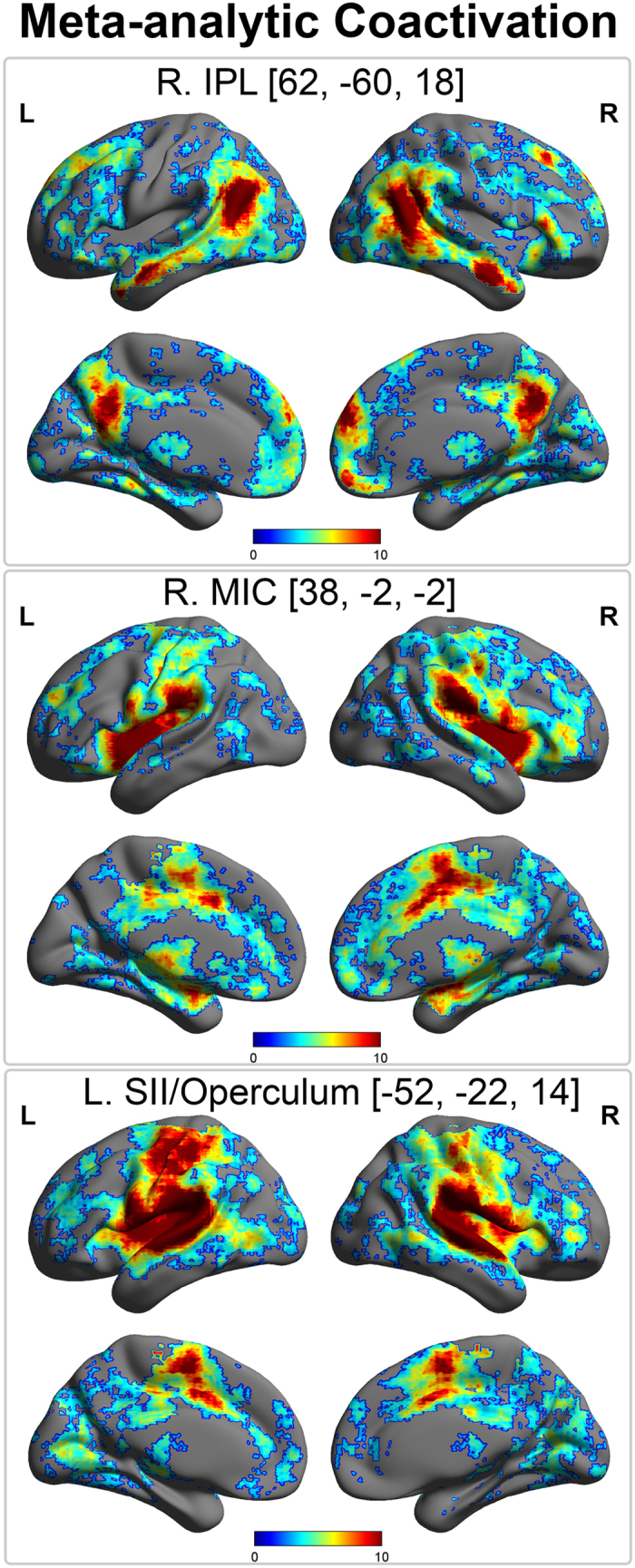
Neurosynth meta-analytic analyses for empathy-related seed regions, showing areas that exhibit significant co-activation with empathy-related seeds identified by our voxel-based quantification analysis. Top panel, the area of the right IPL in which myelination markers (R_1_) covaried positively with Empathic Concern and negatively with Fantasy scores is part of a canonical default mode network including posterior cingulate and medial prefrontal cortex. Quantitative reverse inference revealed that this location was predictive of theory-of-mind related functional terms (see Table 3). Middle and bottom panel, MIC and SII seeds (whose microstructure related to affective and cognitive empathy, respectively) are part of an affective-salience network including dorsal-midcingulate, insula, amygdala, and primary somatosensory cortex. Interestingly, reverse inference revealed that the former was mostly predictive of affective salience (e.g., pain, disgust, intensity), whereas the latter was predictive of somatosensory touch. Images created by projecting Neurosynth meta-analytic z-values onto a smoothed, inflated cortical template using BrainNet Viewer; colorbar indicates z-value range which have been thresholded from 0 to 10 for visualization purposes. Coactivation map adjusted for multiple comparisons, *p*FDR < 0.01, see *Methods* for more details.

Finally, we conducted a meta-analytic co-activation analysis of our peak left IPL, right middle insula (MIC), and left operculum (SII) results. To do so, we used the publically available meta-analytic database Neurosynth (Neurosynth.org) with the peak IPL, MIC, and SII coordinates as seed regions. This provided meta-analytic maps of areas showing significant (false-discovery rate *p* < 0.01) co-activation with our seed regions, as well as a list of associated psychological/functional terms. By assessing the posterior probability of a term appearing in a paper, given a pattern of activation (i.e., p(term | activation)), this analysis constitutes a data-driven reverse inference of the psychological functions implied by an observed brain response^43^,^44^. To summarize these results, all associated terms with a non-zero reverse inference Z-score were sorted by their posterior probability. The top ten terms for each coordinate are presented in Table 3; Fig. 3 depicts the meta-analytic maps for each location.

This analysis revealed that the region of the right IPL which showed correlations with the EC and FS subscales was a part of the default-mode network, with strong co-activation in the mPFC, PCC, and lateral temporal lobes^45^,^46^. Functionally, this location was strongly related to theory of mind (example top terms: “TOM”, “beliefs”, “mental state”). In contrast, both the right MIC and left SII regions implicated in affective and cognitive empathy, respectively, showed strong co-activation throughout the ventral attention or salience network, in particular the dorsal-midcingulate, bilateral insula, somatosensory cortex, and amygdala^47^,^48^. Interestingly, the primary distinction between the two networks was that the left operculum showed greater co-activation with bilateral primary somatosensory areas. Indeed, inspection of the associated meta-analytic terms revealed that whereas our SII region was primarily involved in touch (example top terms: “somatosensory”, “tactile”, “heat”), the right insula region was more involved in affective states and salience (“noxious”, “intense”, “disgust”). These results suggest that the microstructural brain features underlying distinct aspects of empathic skills relate to unique brain networks and functional characteristics. See Table 3 and Fig. 3 for summary of our meta-analytic results.

## Discussion

The present study aimed to map the microstructural brain features underlying individual differences in cognitive and affective trait empathy, as measured by the IRI. To do so we utilised MPM, a quantitative MR imaging technique, to map markers of local myeloarchitectural integrity and iron for each subscale of the IRI, and examined these maps for individual differences using multiple regression VBQ analyses. Our findings show that individual differences in measures of trait empathy are related to underlying microstructural features of brain areas previously associated with empathetic, affective, and bodily (e.g., interoceptive and nociceptive) signal processing, i.e., the insular and somatosensory cortices^49^. These results indicate the potential of quantitative neuroimaging to reveal novel biomarkers, and may inform future research into the clinical and developmental underpinnings of trait empathy.

Following previous volumetric findings using the IRI^25^,^26^,^27^, our VOI analysis suggests myelination levels in the IPL, as indexed by the R_1_ map^50^, are positively related to scores on the Empathic Concern sub-scale (capturing affective empathy), but negatively relate to scores on the Fantasy sub-scale, related to cognitive empathy. While some research suggests the IPL plays a prominent role in affective empathy via emotional contagion^51^, it has also been implicated in cognitive aspects of empathy such as mentalizing and theory of mind^52^. Furthermore, our meta-analytic results also indicate a role for the IPL in these cognitive aspects, as part of the default mode network. Thus, the VBQ results obtained here could suggest myelination levels in the IPL underlie one’s tendency to exhibit either affective or cognitive aspects of empathy.

The MT map captures the effects of macromolecules^42^, predominantly myelin content^53^,^54^, but that may also be associated with the cell membranes of oligodendrocytes and microglia^55^, which facilitate myelination and overall myeloarchitectural integrity. In accordance with our results, a previous study using the IRI to examine trait empathy levels in patients with neurodegenerative diseases, showed an association between empathy deficits and decreased volume in the right caudate^56^. Previous studies have shown that apparent changes in volume can be explained by changes in specific underlying quantitative parameters^30^,^31^. Thus, the previously observed reduction in caudate volume, using VBM, may be explained by the reduction of myeloarchitectural integrity observed here. Although we conducted a VBM analysis using synthetic MPRAGE-like images, this volumetric analysis proved inconclusive. We do not interpret this as evidence against the involvement of these areas but rather as further evidence of the complex relationship between microstructural and volumetric measurements. Future studies should combine VBM-optimized structural images with MPM sequences to evaluate the role of brain microstructure in mediating volumetric effects. Nevertheless, our findings extends previous results from VBM studies associating GM volume in the insula and caudate with affective empathy and personal distress^25^,^26^,^27^.

Expanding previous findings, VOI and whole-brain analyses revealed a negative association between Personal Distress and MT in the middle and anterior insula (AI). In general, these areas are important for emotional regulation and the integration of top-down and bottom-up visceromotor control signals^57^,^58^. Previous research also demonstrates a role for the insula in social emotions, generally^59^, and affective empathy, specifically^3^. For example, a recent meta-analysis of 32 fMRI studies on empathy for pain indicates a consistent pattern of activations in AI in response to observation of others experiencing pain^60^. Moreover, such neural activation is modulated by the observer’s trait empathy scores as captured by the IRI, suggesting that the ability to empathize varies between individuals and that this variation is reflected in neural activations^16^,^23^,^61^. In terms of brain anatomy, individual differences in affective empathy have also been related to neuroanatomical structural characteristics of the insula. For example, several findings suggest that increased GM volume in the insula is positively associated with the tendency to share others’ emotional experiences^25^,^26^,^27^. Our results are thus consistent with the general role of the insula in emotional and affective empathy. Indeed, our meta-analyses indicated that the region associated with Personal Distress is involved in affective processing, as part of the salience network. More specifically, the observed negative relationship between insula myelination and Personal Distress may depend on the role of the insula as an integrative emotional hub^57^,^58^. This could arise either because reduced insula myelination dysregulates incoming visceral and somatic inputs (e.g., a bottom-up effect), or disrupts the ability to cognitively re-appraise or regulate responses to other’s emotions. Future research may thus benefit from combining VBQ measures of insula microstructure with measures of trait arousal, interoception, and/or emotional regulation to further elucidate this finding.

With regards to cognitive empathy, the secondary somatosensory cortex (SII), as part of the parietal operculum^62^, has been previously implicated in the perception and processing of pain^63^,^64^, as well as in social perception^65^. For example, Jackson and colleagues^66^ found that self-perspective, a component of perspective taking, was associated with increased BOLD response in SII. Similarly, imagined experience of another person’s pain has been associated with activity in SII^67^. Our findings are thus consistent with these, suggesting that SII plays a general role in Perspective Taking, demonstrating that individual differences in iron concentration correlate with this subscale^4^.

Iron plays a crucial role in the developing brain, where it is used by oligodendrocytes to drive ATP-synthesis to create and maintain myelin sheaths^68^,^69^. Disruption of this process by nutritional deficiency during foetal, childhood, or adolescent development has been linked to behavioural and neuronal deficits^70^,^71^,^72^. In the developing brain, iron is closely related to neuro-inflammation, potentially caused by stress and nutrition^73^. However, iron also accumulates in the brain throughout the lifespan, contributing to neurodegeneration and demyelination in the aging population^35^,^74^. Given the complex inter-relationship of brain iron content and myeloarchitecture in neural development, the neurobiological interpretation of these results will depend upon future research, examining how different environmental and genetic factors mediate the observed relationship between brain iron and empathy. For example, as it has been suggested that socioeconomic and other developmental stressors play a role in one’s ability to empathise^75^,^76^, it will be interesting to determine how the contribution of early life trauma to brain iron impacts traits such as empathy, which are important factors of social-functioning and well-being^77^,^78^.

Finally, while we find significant correlations between brain microarchitecture and trait empathy, some possible limitations of this study should be noted. All neuroanatomical studies requiring spatial normalisation are potentially susceptible to residual registration errors and partial volume effects. To minimise these biases, we have used the DARTEL algorithm for inter-subject registration, which has been demonstrated to provide maximally accurate registration^79^, and also used the VBQ normalisation procedure, which minimizes partial volume effects introduced by smoothing during normalisation^34^. Importantly, our effects are far from regions that are typically problematic for voxel-based segmentation and normalization (see Supplementary Figures 4 and 5). Additionally, although the IRI is a well-validated measure of trait empathy, the interpretation of self-report scales can be confounded by demand characteristics and other biases. Future research will thus benefit from combining multimodal brain imaging with both implicit and explicit measures of state and trait empathy, to fully tease apart the relationship of trait empathy and individual differences in neuroanatomy. Nevertheless, our study demonstrates for the first time the sensitivity of quantitative MRI biomarkers to IRI-based measures of trait empathy, extending previous functional and volumetric findings. Furthermore, as our measures are quantitative in nature, they are less susceptible to spurious differences in image acquisition and can be directly compared to future results, improving the robustness of future research.

To conclude, using a quantitative multi-parameter mapping approach we found that insular and SII myeloarchitecture and iron level markers underlie individual differences in empathic ability. Our results indicate that reduced SII iron content is associated with higher scores on the Perspective Taking sub-scale, whereas reduced mid-insula myeloarchitectural integrity is associated with lower scores on the Personal Distress sub-scale. Future research looking into the microarchitecture underpinnings of empathy, and its dysfunction in some populations, may build on these findings to better understand the neurobiological and developmental factors driving social skills.

## Methods

### Participants

We recruited 48 healthy participants (29 female) from the University College London and surrounding community using broadcast emails to a local database. Our inclusion criteria required that all participants be between the ages of 20–40 years, right handed, and free from any mental or physical illness. All participants had no prior history of surgery or neurological disorder. Informed consent was obtained from all participants, and in accordance with the declaration of Helsinki the UCL ethics committee approved all procedures.

### Study Design & Overview

All participants attended the Wellcome Trust Centre for Neuroimaging in two sessions. In the first 1-hour appointment, participants underwent MRI scanning. In the second 1-hour appointment, participants completed the IRI questionnaire. Neuroimaging data were acquired using a 30-minute multi-parameter mapping (MPM) sequence (see *MPM Acquisition*, below), during which participants silently viewed a muted nature documentary to promote stillness. During the behavioural session, participants completed the IRI, as well as other measures of auditory perception and metacognitive ability (data not reported here).

### Empathy Questionnaire – IRI

The Interpersonal Reactivity Index (IRI) is an extensively validated questionnaire of self-reported empathic behaviour, which includes 4 individual subscales measuring perspective taking, fantasy, empathy concern, and personal distress^4^,^80^. Perspective Taking measures the propensity to think from another perspective (i.e., theory of mind), whereas Fantasy measures the participant’s ability to imagine themselves in fictional situations. Empathic Concern and Personal Distress are both closely related to affective response, but with a focus on feelings of compassion for others in the former versus having aversive emotional feelings (e.g., fear, anxiety) when witnessing another’s pain or anguish in the latter. Here, we focused on the individual subscales, which have previously been shown to predict volumetric measures of neuroanatomy^25^,^56^, have unique heritability^80^, and because some components (i.e., Personal Distress) of the IRI negatively predict social competence^4^. Each subscale consists of seven questionnaire items, measured on a five point Likert scale ranging from 0 (“Does not describe me well”) to 4 (“Describes me very well”). A score ranging from 0 to 28 was possible for each subscale. Participants completed the IRI in a quiet testing room using the Survey Monkey (https://www.surveymonkey.com/) website. All behavioural and demographic data are available for download at Figshare (URL: https://dx.doi.org/10.6084/m9.figshare.3119011.v1).

### Multi-parameter map acquisition, pre-processing, and analysis

#### Overview

Following recent technical developments, it is now possible to perform *in vivo* mapping of neuroimaging markers of biologically relevant quantities with high resolution, whole brain coverage in acceptable scan times^40^,^79^. To do so, we used the newly developed Multi-Parameter Mapping (MPM) protocol^81^ to obtain maps of the percent saturation due to magnetization transfer (MT), longitudinal relaxation rate (R_1_), and effective transverse relaxation rate (R_2_*) with 800 μm isotropic resolution. These images were then used in a voxel-based quantification analysis (VBQ), which applies specialized tissue segmentation and normalization procedures to minimize partial volume effects while optimally preserving the quantitative values^34^. Finally, we estimated correlations between derived markers of myelination (MT, R_1_) and iron concentration (R_2_*) in multiple regression analyses while controlling for a variety of potential confounders.

#### MPM Data Acquisition

All imaging data were collected on a 3T whole body MR system (Magnetom TIM Trio, Siemens Healthcare, Erlangen, Germany) using the body coil for radio-frequency (RF) transmission and a standard 32-channel RF head coil for reception. The whole-brain quantitative MPM protocol consisted of 3 spoiled multi-echo 3D fast low angle shot (FLASH) acquisitions and 2 additional calibration sequences to correct for inhomogeneities in the RF transmit field^81^,^82^,^83^.

The FLASH acquisitions had predominantly proton density (PD), T1 or MT weighting. The flip angle was 6° for the PD- and MT-weighted volumes and 21° for the T1-weighted acquisition. MT-weighting was achieved through the application of a Gaussian RF pulse 2 kHz off resonance with 4 ms duration and a nominal flip angle of 220°. The field of view was 256 mm head-foot, 224 mm anterior-posterior (AP), and 179 mm right-left (RL). Gradient echoes were acquired with alternating readout gradient polarity at eight equidistant echo times ranging from 2.34 to 18.44 ms in steps of 2.30 ms using a readout bandwidth of 488 Hz/pixel. Only six echoes were acquired for the MT-weighted acquisition in order to maintain a repetition time (TR) of 25 ms for all FLASH volumes. To accelerate the data acquisition, partially parallel imaging using the GRAPPA algorithm was employed with a speed-up factor of 2 in each phase-encoded direction (AP and RL) with forty integrated reference lines.

To maximise the accuracy of the measurements, inhomogeneity in the transmit field was mapped using the 2D STEAM approach described in Lutti *et al*.^82^, including correcting for geometric distortions of the EPI data due to B0 field inhomogeneity. Total acquisition time for all MRI scans was less than 30 mins.

#### Parameter Map Estimation and Voxel-Based Quantification (VBQ)

All images were pre-processed and analysed using SPM12 (version 12.2, Wellcome Trust Centre for Neuroimaging, http://www.fil.ion.ucl.ac.uk/spm/) and bespoke tools implemented in the voxel-based quantification (VBQ) toolbox version 2e^33^,^34^, implemented in MATLAB (Mathworks Inc, version R2014a).

To create the quantitative maps, all weighted volumes were co-registered to address inter-scan motion. Maps of R_2_* were estimated from the gradient echoes of all contrasts using the ordinary least squares ESTATICS approach^84^. The image data for each acquired weighting (PDw, T1w, MTw) were then averaged over the first six echoes to increase the signal-to-noise ratio (SNR)^85^. The three resulting volumes were used to calculate MT and R_1_ as described in^42^,^86^ including corrections for transmit field inhomogeneity and imperfect spoiling^87^,^88^,^89^. The MT map depicts the percentage loss of signal (MT saturation) that results from the application of the off-resonance MT pre-pulse and the dynamics of the magnetization transfer^86^.

A Gaussian mixture model implemented within the unified segmentation approach was used to classify MT maps into grey matter (GM), white matter (WM) and cerebrospinal fluid (CSF)^90^. Diffeomorphic image registration (DARTEL)^91^ was used to spatially normalise individual grey and white matter tissue classes generated from the structural MT maps to a group mean template image. The resulting DARTEL template and participant-specific deformation fields were used to segment and normalise the MT, R_1_ and R_2_* maps of each participant to standard MNI space. We based our normalization of the quantitative maps on the segmentation results of the MT maps because of their greatly improved contrast in subcortical structures, e.g., basal ganglia, and their WM/GM contrast in the cortex being equivalent to T1-weighted images^92^. A 4 mm full-width at half-maximum (FWHM) Gaussian smoothing kernel was applied to the R_1_, MT, and R_2_* maps during normalisation using the VBQ approach, which aims to minimise partial volume effects and optimally preserve the quantitative values^34^. For results visualization, an average MT map in standard space was created from all participants.

#### Analysis – IRI

Each participant’s score on the individual IRI subscales was calculated. As our goal was to perform a multiple regression analysis with all subscales as predictors for the quantitative maps, we first checked linearity assumptions, calculating summary statistics and correlations between each subscale. As previous studies have reported gender differences on the IRI, we also performed independent samples t-tests between our male and female participants for each subscale. All t-tests and correlations were Bonferroni corrected at α = 0.05 and conducted in JASP (beta version 0.7.5).

#### Analysis – Voxel Based Quantification

We performed voxel-based quantification to examine the microstructural correlates of self-reported trait empathy. To do so, we conducted multiple regression analysis with each IRI subscale predicting MT, R_2_*, or R_1_. We also controlled for the potentially confounding effects of age, gender, the gender by Personal Distress interaction (as revealed by our behavioural analysis, see *IRI Results*) and total intracranial volume. Gender was an important control covariate as previous studies have reported gender differences for all IRI subscales^39^, likewise microstructural neuroanatomy varies considerably with age^35^. To extend previous results, we first performed this analysis within a mask of regions previously reported to show structural covariation with trait empathy. This involved the creation of spherical volumes-of-interest (VOIs) in the left and right insula (MNI_XYZ_ = [−42, 18, 0]; [38, 24, −2]; [39, 9, −23]; [30, 8, 5]), anterior/mid-cingulate (MNI_XYZ_ = [−2, 24, 38]; [3, 24, 33]), inferior frontal gyrus (MNI_XYZ_ = [60, 14, 23]), precuneus/posterior cingulate (MNI_XYZ_ = [10, −50, 36]; [−5, −49, 40]), dorsolateral prefrontal cortex (MNI_XYZ_ = [42, 39, 23]; [38, 39, 233]), medial-prefrontal (MNI_XYZ_ = [−1, 47, −4]), ventrolateral prefrontal cortex (MNI_XYZ_ = [50, 23, −1]), somatosensory cortex (MNI_XYZ_ = [48, −16, 54]), and inferior parietal cortex (MNI_XYZ_ = [55, −55, 11]^25^,^26^,^27^,^28^. The resulting mask is available for download at Neurovault (http://neurovault.org/images/18505/).

We then conducted positive and negative t-tests for the correlation of each IRI subscale with MT, R_2_*, or R_1_, using Gaussian random field theory to correct for multiple comparisons at the peak level within the entire VOI mask, peak *p*FWE < 0.05. We also repeated this analysis across the whole brain, correcting for the cluster level family-wise error (FWE), *p*FWE < 0.05, inclusion threshold, *p* < 0.001^93^, non-stationarity corrected^94^. To visualize our effects, we extracted each participant’s y-value from the peak of any significant effect. We then plotted the corresponding IRI subscale versus these values, while controlling for the same covariates as in the whole-brain analysis. All brain results were plotted on an average of our 48 participant’s normalized MT maps, which have excellent visual contrast. To facilitate future comparison studies, T-contrast maps for all IRI subscales are publically available at Neurovault (URL: http://neurovault.org/collections/1263/).

#### Meta-Analytic Coactivation Analyses and Quantitative Reverse Inference

To identify the macroscopic neural networks involved in the regions we found to be related to trait empathy, we performed a meta-analytic coactivation analysis using the Neurosynth website (Neurosynth.org). This technique has been extensively described elsewhere^43^,^44^; here, we focus on the specifics of our analysis. First, our left IPL, operculum, and right mid-insula areas were used as input seed regions. This yielded z-score maps of areas showing significant (*p*FDR < 0.01) co-activation with these coordinates as obtained from N = 72, 189, and 388 automatically indexed fMRI studies reporting activations within 6 mm of these regions, respectively. For visualization, these maps were then plotted on an inflated standard brain using BrainNet Viewer (http://www.nitrc.org/projects/bnv/)^95^ and thresholded at a z-score from 0 to 10 (Fig. 3). This method also provided a list of terms obtained from these studies, and a corresponding z-score and posterior probability for each term indicating the conditional probability of a term being used in a study conditional on activation at the seed voxel (i.e., P(term|activation)). To characterize the function of each seed region, all terms with non-zero z-scores were ordered by their posterior probability, with the top 10 terms presented in Table 2.

## Additional Information

**How to cite this article**: Allen, M. *et al*. Insula and somatosensory cortical myelination and iron markers underlie individual differences in empathy. *Sci. Rep.* **7**, 43316; doi: 10.1038/srep43316 (2017).

**Publisher's note:** Springer Nature remains neutral with regard to jurisdictional claims in published maps and institutional affiliations.

## Supplementary Material

Supplementary Information

## Figures and Tables

**Table 1:** IRI Descriptive Statistics.

	PT	FS	EC	PD
**Mean**	19.00	18.38	19.90	11.96
**Std. Deviation**	5.116	5.648	5.179	4.722
**Skewness**	−0.4358	−0.1194	−0.8231	0.2562
**Std. Error of Skewness**	0.3431	0.3431	0.3431	0.3431
**Kurtosis**	−0.5589	−0.9503	0.2885	−0.7059
**Std. Error of Kurtosis**	0.6744	0.6744	0.6744	0.6744
**Minimum**	8.000	8.000	7.000	4.000
**Maximum**	28.00	28.00	28.00	23.00

PT = Perspective Taking, FS = Fantasy, EC = Empathic Concern, PD = Personal Distress.

**Table 2:** Summary of VBQ Results.

**(A) Summary of VOI Results**									
**Map**	**Region**	**k**	**p**_**FWE**_**peak**	**p**_**U**_**peak**	**T**	**Z**	**x**	**y**	**z**
R_1_ (+EC)	R IPL	38	0.010	<0.001	5.57	4.75	62	−59	18
R_1_ (−FS)	R IPL	14	0.020	<0.001	5.32	4.58	62	−59	18
R_2_* (−PT)	R Insula	74	0.042	<0.001	4.96	4.34	37	19	5
**(B) Summary of Whole Brain Results**									
**Map**	**Region**	**k**	**p**_**FWE**_**cluster**	**p**_**U**_ **cluster**	**T**	**Z**	**x**	**y**	**z**
MT (−PD)	R Insula	1913	<0.001	<0.001	4.88	4.29	39	−4	−2
MT (−PD)	R Caudate	1363	<0.001	<0.001	5.17	4.48	14	9	−5
R_2_* (−PT)	L S2/OP	253	0.032	0.001	5.31	4.58	−52	−22	13

*Note: k = *cluster extent in voxels, T = t-value, Z = z-value, x,y,z = MNI peak coordinates. *p*_FWE_ *p*eak = family-wise peak corrected p-value, *p*_FWE_ cluster = family-wise cluster corrected p-value, *p*U cluster = uncorrected cluster p-value. + or −indicates positive or negative t-contrast, EC = Empathetic Concern, FS = Fantasy, PD = Personal Distress, PT = Perspective Taking. MT = Magnetization Transfer Saturation map, R_2_* = transverse relaxation rate, R_1_ = longitudinal relaxation rate. See *VBQ analysis* and *VBQ Results* for more details.

**Table 3:** Summary of Meta-Analysis and Quantitative Reverse Inference.

Term	Z	P(term|activation)
**Left Inferior Parietal Lobule**		
mind tom	6.76	0.92
valid	7.63	0.92
tom	6.49	0.91
theory mind	7.59	0.9
beliefs	5.27	0.9
structures involved	4.9	0.89
temporoparietal junction	5.96	0.88
mental state	4.98	0.88
attend	4.72	0.88
mapped	4.54	0.88
**Right Middle Insular Cortex**		
noxious	5.68	0.86
seeds	4.97	0.86
somatosensory cortices	5.93	0.85
sensations	4.75	0.85
intense	4.75	0.85
posterior insula	6.04	0.84
disgust	4.91	0.84
experiencing	4.91	0.84
heat	3.87	0.83
nociceptive	3.83	0.83
**Left SII/Operculum**		
sii	12.22	0.91
secondary somatosensory	10.84	0.87
si	8.15	0.85
index finger	6.94	0.85
somatosensory cortices	7.26	0.84
tactile	9.65	0.84
heat	5.94	0.84
contralateral primary	5.87	0.84
stimulated	6.83	0.84
touch	6.33	0.83

Table shows results of Neurosynth quantitative posterior probability for each psychological term given an activation at that region, and associated z-value. Terms are sorted by their posterior probability.
