# Supplementary material for: Insula and somatosensory cortical myelination and iron markers underlie individual differences in empathy
Source: Sci Rep. 2017 Mar 3;7:43316. doi: 10.1038/srep43316 (PMC5335674; doi:10.1038/srep43316)
Supplement: Supplementary Information [file srep43316-s1.doc]

Supplementary information for:

**Insula and somatosensory cortical myelination and iron markers underlie individual differences in empathy**

Micah Allen^†^*_1,2_, Darya Frank^†^_1,3_, James C Glen_1_, Francesca Fardo_4,5,1_, Martina F. Callaghan_2_, Geraint Rees_1,2_

^†^contributed equally to this work.

*corresponding author

**Author Affiliations:**

_1_Institute of Cognitive Neuroscience, UCL

_2_Wellcome Trust Centre for Neuroimaging

_3_School of Psychological Sciences, University of Manchester

_4_Danish Pain Centre, Department of Clinical Medicine, Aarhus University, 8000 Aarhus, Denmark

_5_Interacting Minds Centre, Aarhus University, 8000 Aarhus, Denmark

Contents: two (2) supplementary tables, supplementary methods, supplementary results, and (2) supplementary figures.

| **Supplementary Table 1 – IRI Pearson Correlations** | | | | | | | | | |
| --- | --- | --- | --- | --- | --- | --- | --- | --- | --- |
|  | | **PT** | | **FS** | | **EC** | | **PD** | |
| PT |  | — |  | 0.258 |  | 0.463 | *** | -0.187 |  |
| FS |  |  |  | — |  | 0.483 | *** | 0.213 |  |
| EC |  |  |  |  |  | — |  | 0.163 |  |
| PD |  |  |  |  |  |  |  | — |  |
|  | | | | | | | | | |
| *** p < .001, PT = Perspective Taking, FS = Fantasy, EC = Empathic Concern, PD = Personal Distress. | | | | | | | | | |

| **Supplementary Table 2 – IRI Gender Differences** | | | | | | | | | | | | | |
| --- | --- | --- | --- | --- | --- | --- | --- | --- | --- | --- | --- | --- | --- |
|  | | **t** | | **df** | | **p** | | **Mean Difference**  **(F – M)** | | **SE Difference** | | **Cohen's d** | |
| PT |  | -0.813 |  | 46.00 |  | 0.420 |  | -1.244 |  | 1.586 |  | -1.644 |  |
| FS |  | -0.829 |  | 46.00 |  | 0.412 |  | -1.400 |  | 1.762 |  | -1.676 |  |
| EC |  | -0.521 |  | 46.00 |  | 0.605 |  | -0.811 |  | 1.665 |  | -1.054 |  |
| PD |  | -2.630 |  | 46.00 |  | 0.012 |  | -3.489 |  | 1.241 |  | -5.317 |  |
|  | | | | | | | | | | | | | |
| Student’s t-test. PT = Perspective Taking, FS = Fantasy, EC = Empathic Concern, PD = Personal Distress. | | | | | | | | | | | | | |

**Supplementary methods and analyses**

At the request of a reviewer, we conducted two additional analyses, consisting of volumetric and surface based analysis of IRI correlates. This involved the creation of a post-hoc ‘synthetic’ MPRAGE image, by recombining our quantitative maps. While these results were inconclusive, we strongly caution against interpreting them as failures to replicate previous volumetric effects due to their lack of optimization for segmentation, normalization, and surface reconstruction.

**Image pre-processing: creation of synthetic MPRAGE**

**Image synthesis:** First, two synthetic FLASH volumes were created using the FreeSurfer mri_synthesize routine. Inputs to the routine were scaled quantitative PD and T_1_ (1/R_1_ volumes), with removal of a small number of negative and very high values produced by estimation errors. For the first synthetic image, default FreeSurfer contrast parameters were specified. The second synthetic image was produced using the same PD and T1 input volumes, with contrast parameters specified (TR = 25 ms; α = 220°; TE = 4 ms). During synthesis, images were ‘conformed’ to 1mm^3^ isotropic resolution in FreeSurfer. Both synthetic images were then further scaled with AFNI 3dcalc; this additional linear scaling yielded image intensity properties closer to the optimal intensity values needed to segment tissue boundaries in FreeSurfer. The image synthesized with default contrast parameters was used as the main input to the FreeSurfer automated processing stream (following further pre-processing steps; see below). The image synthesized with specified contrast parameters was used at a later stage as input to the FreeSurfer Talairach transformation. Finally, a scaled and truncated version of the PD volume was produced with AFNI 3dcalc. This adjusted PD volume was used as input to the skull strip procedure (see below).

**Manual image adjustment:** Each subject’s synthetic image was hand-adjusted using a piecewise linear normalization procedure to linearly ramp intensity values of grey and white matter within isolated regions. Brightness values of voxels within the inferior and medial temporal lobes, temporal pole, long and short insular gyri, and ventro-medial pre-frontal cortex were gently rescaled (< 1.2x). Manual blink comparison between the synthetic volume and the labelled white matter surface was used to compare adjustments as each brightening iteration was applied. Care was taken to ensure that manual brightening did not cause grey and white matter to exceed the intensity value bounds specified for those tissue classes in FreeSurfer (grey matter: 50-70; white matter: 100-140). Manually brightened synthetic images were saved and used within the skull strip procedure.

**Skull strip**: Next, the subject’s adjusted quantitative PD volume (see image synthesis) was used as input to a customized skull strip procedure run in Csurf. Briefly, the skull strip procedure removed the skull and regions exterior to it from the image volume, rendering an image of remaining brain tissue (including cerebellum and brainstem). First, an elliptical surface (4th or 5th geodesic subtessellation of an icosahedron) was expanded from inside the PD volume, with expansion of the surface constrained by arrival at low intensity voxels (i.e., those containing CSF and/or the inner surface of the skull). The set of voxels intersecting the faces of the resulting surface was then flood-filled from the outside, thereby constraining the brain volume to the brighter voxels inside the surface region. Using this PD volume as a mask, flood-filled voxels in the volume were used to set the corresponding voxels in the subject’s default-parameter synthetic image to an intensity of zero. The boundaries of the flood-filled voxels within the skull-stripped PD image were then manually adjusted to correct for any local deviations into neural tissue (particularly in regions proximal to paranasal sinuses, prone to susceptibility artifacts). Manual adjustment involved reducing the intensity threshold for cortical grey matter (to a value of 40); the flood-filled boundary was then forced toward voxels below this threshold. Manual adjustment was applied to the synthetic volume; the skull-stripped synthetic volume was used as input

**Voxel-based morphometry**

To assess whether the microstructural effects observed here where related to previously reported volumetric effects, we performed a voxel-based morphometry (VBM) analysis using SPM12, at the request of a reviewer. To do so we created ‘synthetic’ MPRAGE images by recombining our quantitative images into a single map (see above). Following established protocols, scans were then segmented into gray matter, white matter, and CSF in native space. The DARTEL (diffeomorphic anatomical registration through exponentiated lie algebra) algorithm (Ashburner, 2007) was used for normalization to increase the accuracy of inter-subject registration, by aligning and warping the gray matter images to an iteratively improved template. The DARTEL template was then registered to the Montreal Neurological Institute stereotactic space, and the gray matter images modulated such that their original tissue volumes were preserved. Images were smoothed using an 8mm full-width at half-maximum Gaussian kernel.

The resultant pre-processed images were then analyzed in a multiple regression design matrix to identify brain regions whose volume correlated with IRI subscales, similar to our VBQ analyses, age, and gender, and the gender by PD subscale interaction were included as control covariates. Proportional scaling was used to account for variability in total intracranial volume across participants. A binary gray matter mask (> 0.3) was generated from the average of all subjects normalized, smoothed gray matter segments, to exclude clusters outside the brain and limit the search volume to voxels likely to contain gray matter.

We examined the positive and negative t-maps for each subscale, in both a whole brain FWE-cluster corrected (non-stationarity corrected, inclusion threshold p < 0.001) analysis and a FWE-peak corrected VOI analysis using the same mask of a priori regions as in our VBQ analysis.

**Standard Deviation Mapping for MT and R2***

To address possible concerns regarding image segmentation and normalization images, we also created an average standard deviation map for all MT and R2* gray matter segments separately, using the SPM Image Calculator. We then overlaid the binary cluster mask for each of our whole-brain effects to determine whether effects overlapped with regions of high inter-subject variation (indicating a greater chance of registration error).

**Supplementary Results**

**VBM Analysis**

No results surviving either whole brain or small volume corrections where found. Inspection of the Any Effects F-contrast (e.g., eye(4)) revealed that IRI subscales show minor covariation with brain volume in the posterior and anterior midline cortices, insula, parietal areas, and cingulate (see supplementary figure 1 and 2 below). Additionally, at a low threshold of T > 2 positive covariation with the PD subscale and brain volume could be seen in the middle-anterior insula (supplementary figure 3, below).


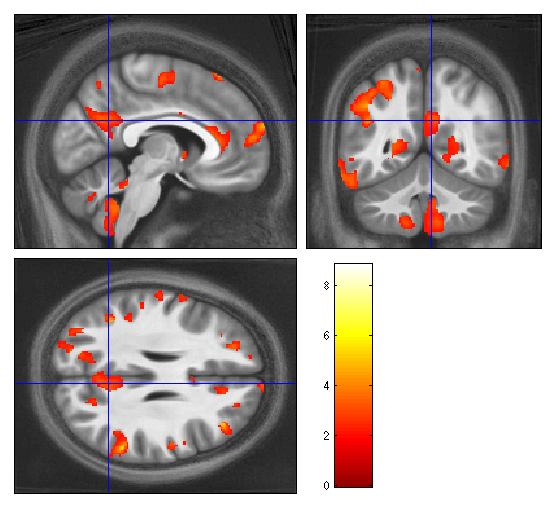


Supplementary figure 1. Although no effects survived either whole brain or VOI correction, at low threshold volumetric variation with the IRI can be seen in midline, parietal, and insula cortices. Uncorrected any effects (eye(4), over 4 subscales) F-contrast, plotted at F > 2.0 for visualization purposes. Colorbar depicts F-value at each voxel.


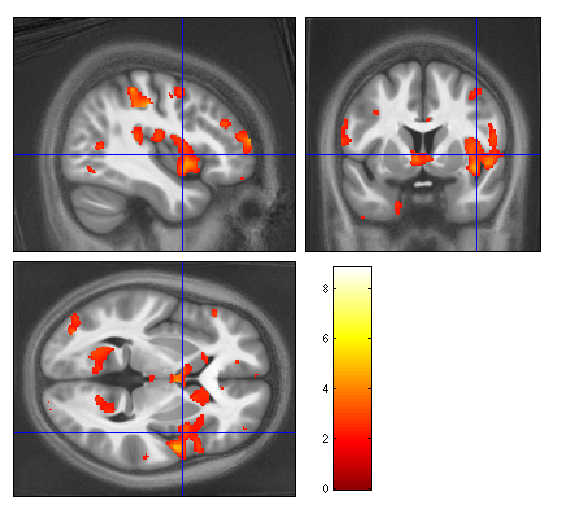


Supplementary figure 2. Although no effects survived either whole brain or VOI correction, at low threshold volumetric variation with the IRI can be seen in midline, parietal, and insula cortices. Uncorrected any effects (eye(4), over 4 subscales) F-contrast, plotted at F > 2.0 for visualization purposes. Colorbar depicts F-value at each voxel.


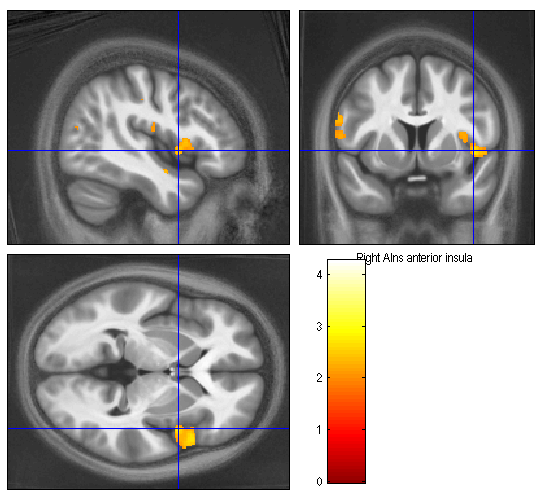


Supplementary figure 3. Although no effects survived either whole brain or VOI correction, at low threshold positive volumetric variation with the PD subscale can be seen in the middle-anterior insula. Uncorrected PD > 0 T-contrast, plotted at T > 2.0 for visualization purposes. Colorbar depicts F-value at each voxel.


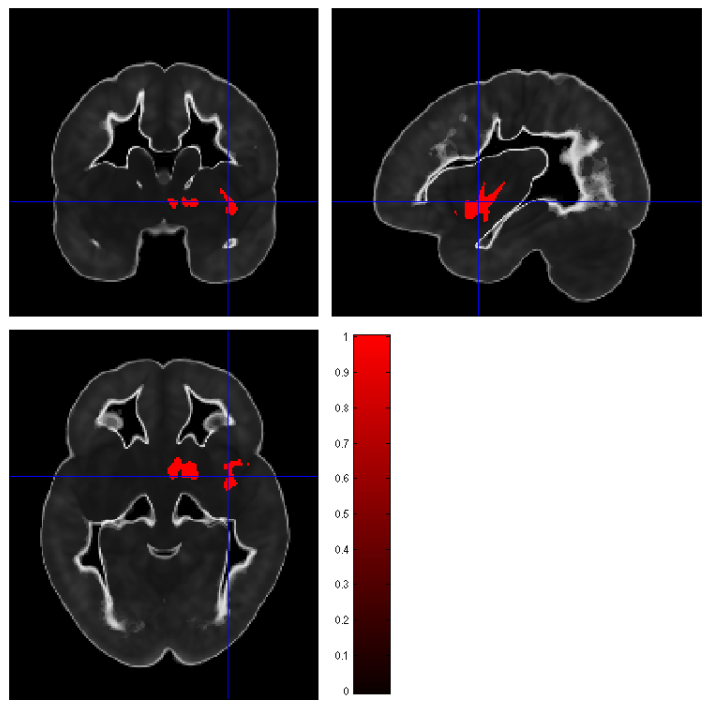


Supplementary Figure 4: MT – PD negative effect (binary mask) overlaid on standard deviation of all MT gray matter segments. Areas that can be problematic for segmentation and normalizaiton (e.g., vascular and white matter boundaries, brain periphery) are far from our obtained effects.


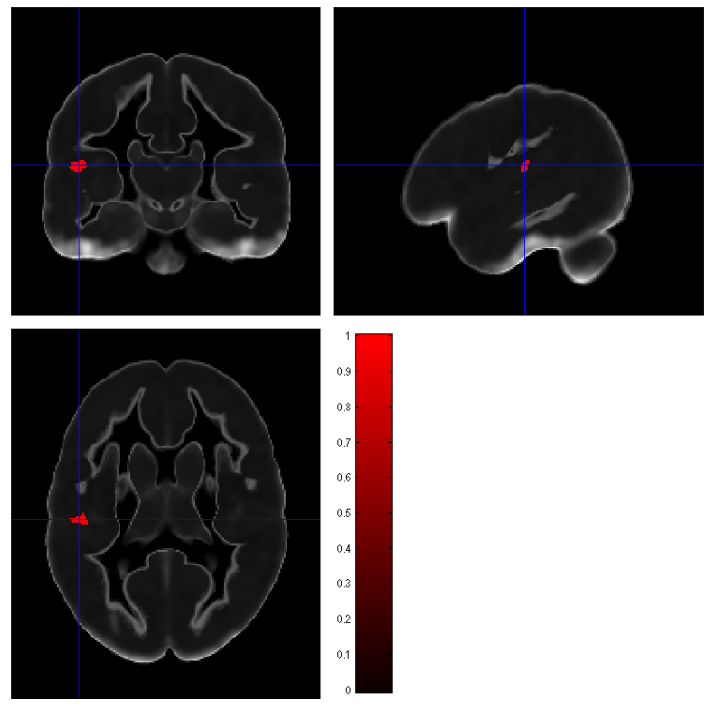


Supplementary Figure 5: R2* – PT positive effect (binary mask) overlayed on standard deviation of all r2* gray matter segments. Areas that can be problematic for segmentation and normalization (e.g., vascular and white matter boundaries, brain periphery) are far from our obtained effects.
